# A study on the correlation between the dewetting temperature of Ag film and SERS intensity

**DOI:** 10.1038/s41598-017-15372-y

**Published:** 2017-11-07

**Authors:** Jiamin Quan, Jie Zhang, Xueqiang Qi, Junying Li, Ning Wang, Yong Zhu

**Affiliations:** 1 The Key Laboratory of Optoelectronic Technology and System, Education Ministry of China, Chongqing University, chongqing, 400044 China; 2 College of Optoelectronic Engineering, Chongqing University, chongqing, 400044 China; 3 College of Chemistry and Chemical Engineering, Chongqing University, chongqing, 400044 China

## Abstract

The thermally dewetted metal nano-islands have been actively investigated as cost-effective SERS-active substrates with a large area, good reproducibility and repeatability *via* simple fabrication process. However, the correlation between the dewetting temperature of metal film and SERS intensity hasn’t been systematically studied. In this work, taking Ag nano-islands (AgNIs) as an example, we reported a strategy to investigate the correlation between the dewetting temperature of metal film and SERS intensity. We described the morphology evolution of AgNIs on the SiO_2_ planar substrate in different temperatures and got the quantitative information in surface-limited diffusion process (SLDP) as a function of annealing temperature *via* classical mean-field nucleation theory. Those functions were further used in the simulation of electromagnetic field to obtain the correlation between the dewetting temperature of Ag film and theoretical analysis. In addition, Raman mapping was done on samples annealed at different temperatures, with R6G as an analyte, to accomplish the analysis of the correlation between the dewetting temperature of Ag film and SERS intensity, which is consistent with the theoretical analysis. For SLDP, we used the morphological characterization of five samples prepared by different annealing temperatures to successfully illustrate the change in SERS intensity with the temperature fluctuation, obtaining a small deviation between the experimental results and theoretic prediction.

## Introduction

Surface-enhanced Raman spectroscopy (SERS) as a powerful analytical technique for ultrasensitive chemical or biochemical analysis has gained much attention since its discovery 40 years ago, because it can produce several orders of magnitude enhancement in Raman signals^1,2^. It is widely accepted that there are two mechanisms to describe the overall SERS effect: the electromagnetic (EM) effect and the chemical effect^3^. After decades of debate, it is now generally agreed that the dominant contributor to most SERS processes is the EM mechanism^4,5^. Specifically, under appropriate circumstances, SERS enhancements as large as 10^14^ can be achieved, in which at least 8–10 orders of magnitude can arise from the EM effect, while the enhancement factor due to the chemical effect is only 10^1^–10^2^ times. EM mechanism is based on the enhancement of the local electromagnetic field, while the chemical mechanism is based on the charge transfer between the adsorbed molecules and the metal surface^6^. The strong electromagnetic field enhancement near metallic nanostructures is the excitation of the localized surface plasmon resonance (LSPR) on the metal surface. Thus, the analytical applications of SERS technique depend strongly on the LSPR properties of the nanostructured metal. The strength of LSPR on the metal surface is determined by the frequency of excitation light and the surface roughness of substrates^7^. By controlling the shape (surface morphology), size, and the spacing between nanoparticles, we can tune the LSPR to obtain an optimized SERS signal from the metal nanostructures at the target wavelength. Among those factors, the surface morphology and the inter–particle spacing are particularly important. Firstly, it has been found that the ultra-samll distance between the nanoparticles (in the region of a few nanometers) can enormously enhance the Raman signals of analyte molecules^8^. Secondly, due to the surface plasmon polarization (SPP) of the high curvature surface of the nanostructures such as tips and sharp edges, strong SERS enhancement is established according to the experimental and simulation results^9^.

In practical application, a SERS-active substrate with a large area, high sensitivity, good reproducibility, and a simple fabrication process is desirable^10,11^. However, a practical SERS substrate that combines all the excellent properties mentioned above could hardly be found. The thermally dewetted AgNIs have been numerous studied in SERS due to its overall performance with good reproducible, passable sensitivity, cheap cost, simple fabrication process and easy to be fabricated in a large area^12,13^. For the metal nano-islands sample, it has been demonstrated that a SERS substrate with remarkable enhancement (*EF* = 0.8 × 10^9^) or excellent reproducibility (RSD = 12.1%) can be obtained *via* the second-deposition method (by depositing an additional Ag film on a silicon substrate)^13^. Moreover, the application of metal nano-islands in SERS has been greatly expanded. AgNIs-graphene oxide exhibits great potential for diverse aromatic molecules sensing^14^. A structure of AgNIs-graphene-AgNIs has achieved an ultrasensitive SERS detection with a limit of down to 10^−13^ M^15^. All those studies are based on the dewetting of metal film affected by the dewetting condition. But the research of correlation between the dewetting temperature of metal film and SERS intensity is only limited to experience and experiment. There is no detailed research to determine the correlation between the dewetting temperature of metal film and SERS intensity.

**Figure 1:**
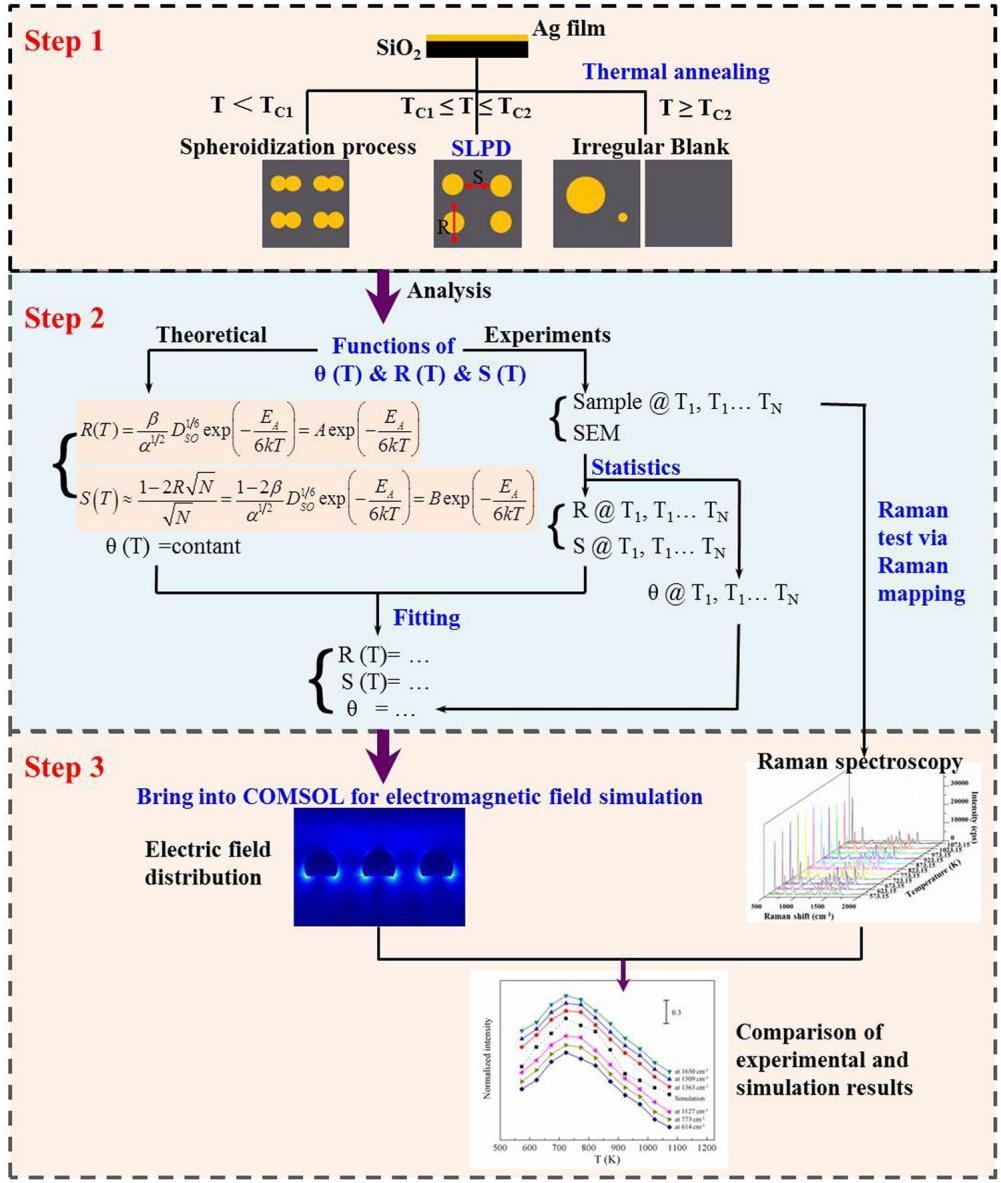
The “three-step” approach.

In this work, we provided a “three-step” approach to investigate the correlation between the dewetting temperature of metal film and SERS intensity, shown in Fig. 1. First, the mean diameter, mean surface-to-surface spacing and contact angle of the thermally dewetted AgNIs were quantified as a function of the annealing temperature T *via* the classical mean-filed nucleation theory, based on the image analysis of scanning electron microscopy (SEM) of AgNIs in SLDP. Subsequently, these functions were brought into the COMSOL Multiphysics to obtain the correlation between LSPR effect and the dewetting temperature. Finally, using the R6G as the probe molecule to conduct SERS measurements, we found there was a good consistency between the simulation and experimental results.

## Results

### Characterization of scanning electron microscope

**Figure 2:**
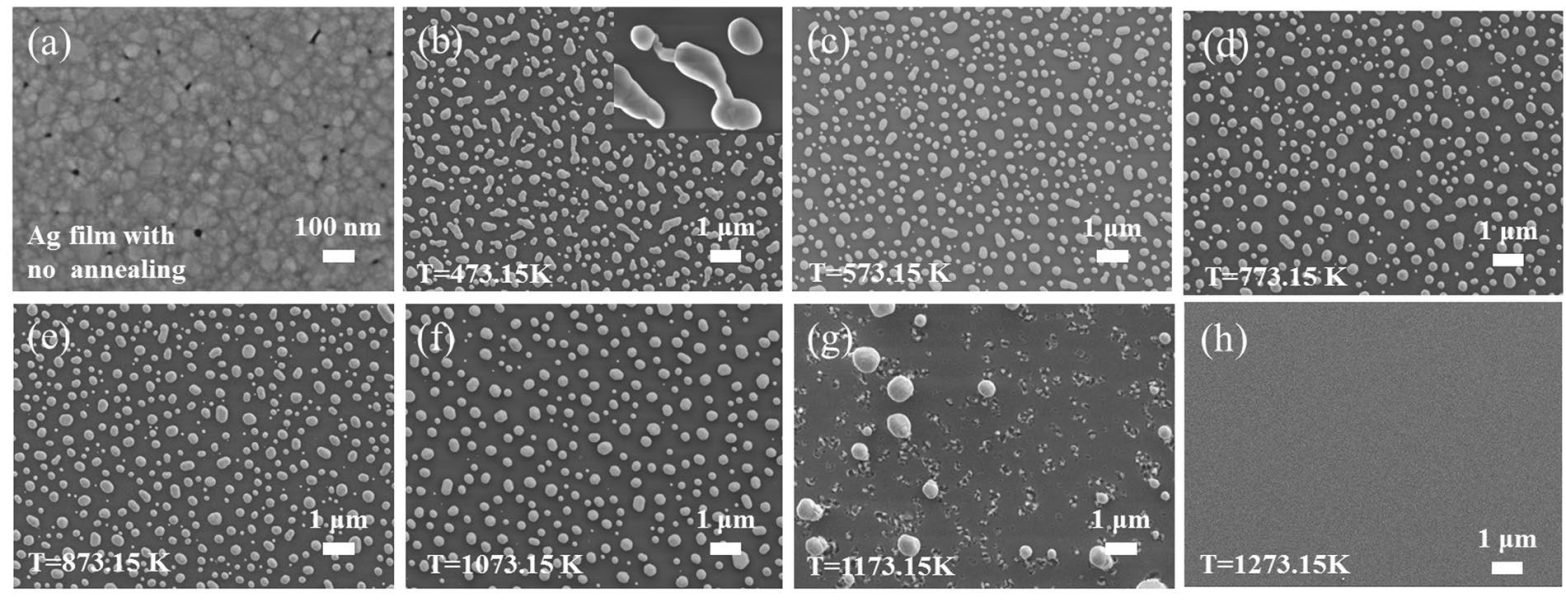
Representative SEM images of (**a**) Ag film (with no annealing), with additional annealing for 1 h at (**b**) 473.15 K, (**c**) 573.15 K, (**d**) 773.15 K, (**e**) 873.15 K, (**f**) 1073.15 K, (**g**) 1173.15 K and (**h**) 1273.15 K.

Fig. 2a is the SEM image of the 20-nm-thick Ag film deposited on the SiO_2_ substrate *via* vacuum thermal evaporation. The Ag film is quasi-continuous, since the thickness of 20 nm is not thick enough to form a perfectly continuous film. Atoms deposited from the vapor phase undergo a series of kinetic processes, including thermal accommodation onto the substrate, surface diffusion of the atoms on the surface, dimer formation to initiate nucleation, and island formation and growth^16^. As more and more atoms are deposited, small islands may grow, contact with each other, and then fully coalesce (i.e., two islands merge into larger, but still compact islands)^17,18^. The deposited layer can be considered as comprising numerous nanoscale Ag crystalline grains. In the entire quasi-continuous Ag film layer, there are tight connections between Ag crystalline grains.

However, there is a difference under additional annealing process. The deposited film will be in a metastable configuration that tends to equilibrate^19^, once subjected to temperature by annealing, and the difference of surface free energy between Ag and SiO_2_ will induce the dewetting of Ag film on SiO_2_^20^. Dewetting is generally initiated by heterogeneous void formation during annealing, and is driven by excess energy due to high fraction of grain boundaries, interfaces and surfaces, or residual stresses^21^. Experimental data on thin film dewetting have been quantitated in nature, with limited insights on the kinetic mechanisms of void nucleation and void growth processes^22^.

**Figure 3:**
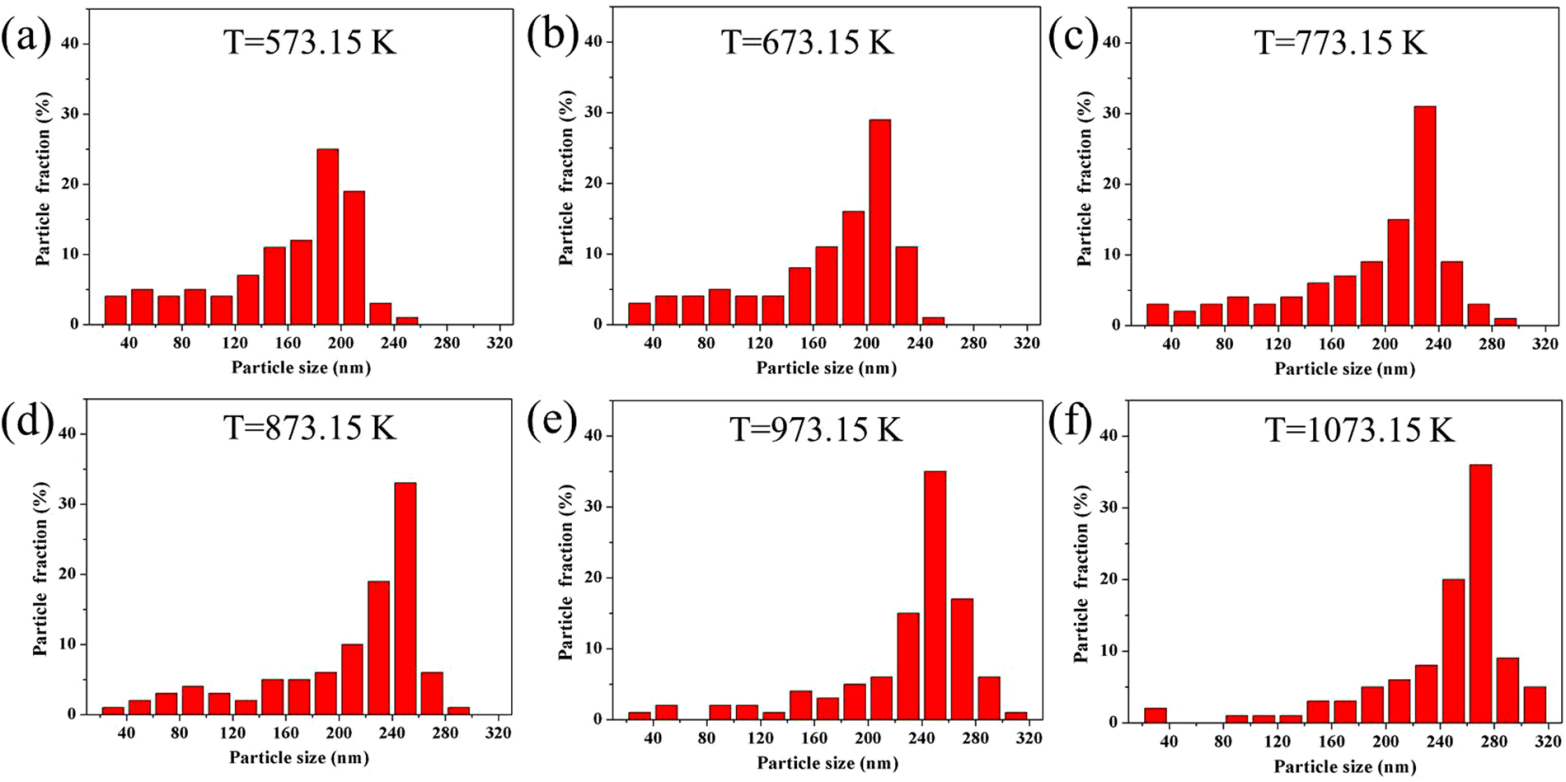
Diameter distribution histogram of AgNIs annealed at (**a**) 573.15 K, (**b**) 673.15 K, (**c**) 773.15 K, (**d**) 873.15 K, (**e**) 973.15 K and (**f**) 1073.15 K.

We show one set of isochronal annealing treated Ag film at some representative temperatures with an initial film height of 20 nm in Fig. 2b∼f. We noticed there were two critical temperatures in our annealing process, which were 573.15 K (T_*c*1_) and 1073.15 K (T_*c*2_), respectively. An analysis was based on the following points:

1. For annealing temperatures below first critical temperature (T_*c*1_ = 573.15 *K*), the thin film is undergoing a transition from quasi-continuous to discontinuous. The connections between the Ag domains are gradually separated under the continuous heating. The Ag atoms diffuse away from the regions of high curvature. Diffusion should continue until an equalized surface is reached^23^. When the annealing temperature reaches T_*c*1_, the resultant equilibrium morphology of a grain would be spherical caps, with the perimeter held at the equilibrium contact angles. According to the second law of thermodynamics, there is a general natural tendency to achieve a minimum of the Gibbs free energy. The whole spheroidization process can be formulation analysis by the minimization of the system’s free energy^24^. Fig. 2b and c show the film morphology annealed at 473.15 K and 573.15 K, respectively. Since the temperature of 473.15 K cannot bring enough energy to the sample to complete the whole spheroidization process, the morphology of AgNIs is not spherical and some connections are not separated (Fig. 2b). But in Fig. 2c, we find spheroidization process is completed. All AgNIs are spherical and there are no connections between AgNIs.
2. When the annealing temperatures are between two critical temperatures, all AgNIs are hemispheric, but the AgNIs surface density N, mean diameter R and mean surface–to-surface distance S are fluctuant with the temperature. We show the SEM and AgNIs diameter distribution histogram of AgNIs annealed at some representative temperatures within two critical temperatures in Fig. 2c∼f and Fig. 3c∼f, respectively. Meanwhile, the R and S are calculated in Table 1. We noticed, that as the temperature increased, the AgNIs surface density N decreased and, correspondently, R and S increased. Moreover, with the increase of temperature, small particles are lessened and the value distribution of AgNIs diameter became more concentrated, which indicated that the substrate becomes more uniform. The changes in the AgNIs morphologies at different temperatures are completed *via* surface-limited diffusion^25,26^, which is analogous to ostwald ripening. This process can be attributed to the varying of surface diffusion coefficient with temperature^27^. For each fixed temperature T, the smaller AgNIs (characterized by a diameter smaller than a critical one) are thermodynamically unstable and they dissolve in atoms. These Ag atoms diffuse on the SiO_2_ surface to be incorporated by AgNIs with diameter larger than the critical one, due thechemical potential difference among different AgNIs^28^. With the increase of temperature, the surface diffusion coefficient increases, which means that an arriving atom has a high probability to find an existing island, rather than to form a new island, resulting in the decrease (increase) of Ag particle density (size). We will focus on this process to investigate the correlation between the dewetting temperature of metal film and SERS intensity. At the same time, we name this process surface-limited diffusion process (SLDP) for more convenient expression.

**Table 1:** Mean diameter (R) and mean surface-to-surface spacing (S) of AgNIs at different temperatures.

	573.15 K	673.15 K	773.15 K	873.15 K	973.15 K	1073.15 K
R (nm)	175.65	194.07	215.31	229.63	237.69	254.74
S (nm)	122.12	139.59	147.71	161.44	170.11	177.15
3. As the temperature rises upon the second critical temperature, the influence of thermal annealing on samples is complex. First, some of Ag will volatilize from substrate at high temperature, and this phenomenon will be more obvious with the increase of the temperature^29^. Second, SiO_2_ will soften under the high temperature. If the softening is too severe, SiO_2_ couldn’t provide stable interface for the diffusion of Ag atoms. Fig. 2g shows the thermally dewetted AgNIs in 1173.15 K is much bigger and sparser than that below 1173.15 K, which obviously doesn’t follow the rules in SLDP. Moreover, when the annealing temperature reaches 1273.15 K, there is even no nanostructure on the surface of the substrate (shown in Fig. 2h). In order to investigate the whereabouts of the AgNIs, area energy dispersive spectrometer (EDS) analysis was used to confirm the presence of silver element. An analysis of EDS shown in Fig. 4 reveals no silver element on the whole substrate. For AgNIs, the melting temperature is lower than the bulk melting point^30^. Thus, we conclude that 1273.15 K is high enough for the atom of our AgNIs to volatilize from the substrate, which leads to the absence of nanostructure on the substrate.

**Figure 4:**
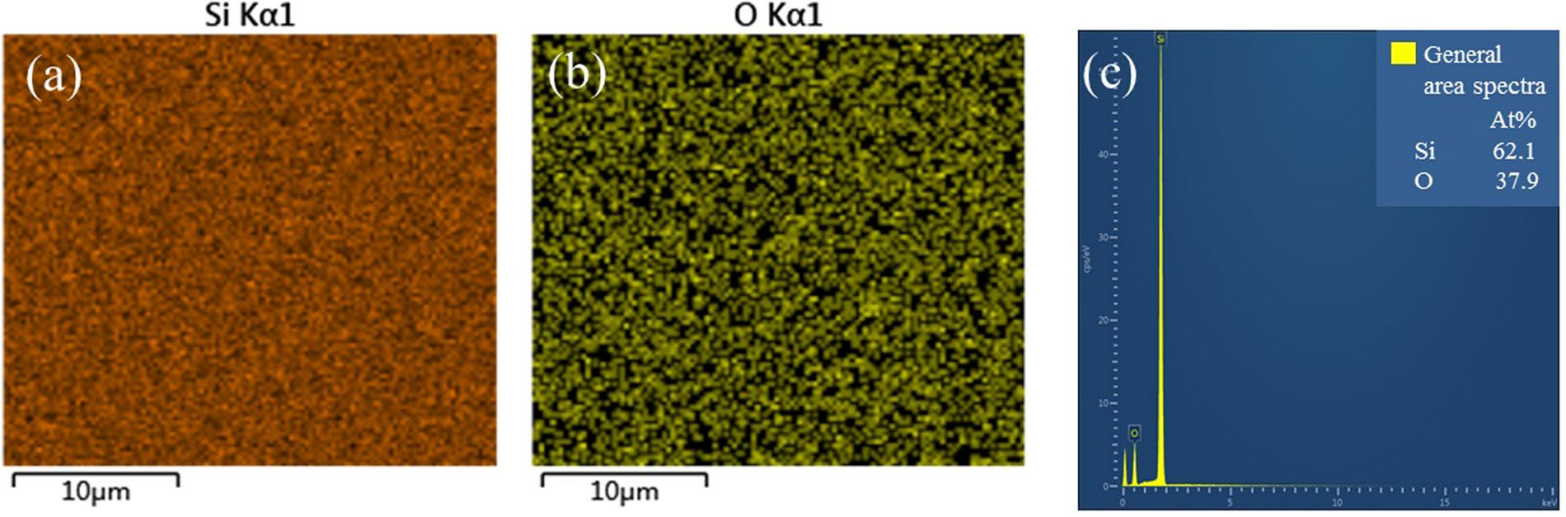
Results of EDS analysis for sample annealed at 1273.15 K. (**a**∼**b**) The distribution diagram of element for Si and O, respectively; (**c**) general area spectra of elements concentration.

### Quantitative analysis

In our work, we focus on the correlation between the dewetting temperature of Ag film and SERS intensity in SLDP. Thus, we try to get the quantitative information about the morphology evolution of AgNIs in SLDP as a function of the annealing temperature. There are at least three parameters needed to express all morphological information of AgNIs, which are R, S and *θ*, respectively. Thus, we carry out the following analysis to obtain the quantitative information of those three parameters.

For the quantitative analyses, we consider the relationship between the surface diffusion coefficient (*D*_s_) and the temperature (T), which can be described by^18,31^

1 $${{D}}_{{S}}({T})={{D}}_{{S}0}{\exp }(-\frac{{{E}}_{{A}}}{{KT}})$$

where *D*_S0_ is the pre-exponential factor, *E*_*A*_ is the activation energy for the diffusion process, K is the Boltzmann constant, and T is the absolute temperature. Based on the relationship between the surface diffusion coefficient (*D*_*S*_) and the temperature (T), we could evaluate R and S for each annealing temperature.

According to the classical mean-field nucleation theory, the AgNIs density can be described by ${N}\propto {(1/{{D}}_{{S}})}^{1/3}$ for isotropic surface diffusion^32^. Thus, after introducing the proportionality constant α, we can write

2 $${N}({T})=\frac{{\alpha }}{{{D}}_{{S}0}^{1/3}}{\exp }(-\frac{{{E}}_{{A}}}{{KT}})$$

In SLDP, the relationship between R and N can be described as ${R}\propto 1/{{N}}^{1/2}$ ^31,33^. Thus, after introducing the proportionality constant *β* and A, we can write

3 $${R}({T})=\frac{{\beta }}{{{\alpha }}^{1/2}}{{D}}_{{S}0}^{1/6}{\exp }(-\frac{{{E}}_{{A}}}{{KT}})={A}\,{\exp }(-\frac{{{E}}_{{A}}}{{KT}})$$

Meanwhile, to find the relation between S and T, if we suppose the AgNIs disposed in an ordered squared arrays of side L, the linear space of such a side occupied by AgNIs is $2{RL}\sqrt{N}$, the free linear space is ${SL}(\sqrt{{N}}-1)\approx {SL}\sqrt{{N}}$. So that, we can get the equation ${L}\approx 2{RL}\sqrt{{N}}+{SL}\sqrt{{N}}$. For the S, introducing the proportionality constant B, we can write

4 $${S}({T})\approx \frac{1-2{R}\sqrt{{N}}}{\sqrt{{N}}}=\frac{1-2{\beta }}{{{\alpha }}^{1/2}}{{D}}_{{S}0}^{1/6}{\exp }(-\frac{{{E}}_{{A}}}{{KT}})={Bexp}(-\frac{{{E}}_{{A}}}{{KT}})$$

The Eqs 3 and 4 show R and S are a function of T, and the values of three constants (*E*_*A*_, A and B) are undetermined. So, we further carried out a quantitative study on R and S by an image analysis of the thermally dewetted AgNIs.

**Figure 5:**
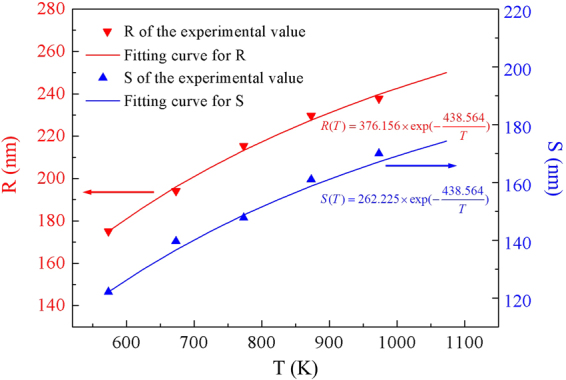
Evolution of mean diameter R and mean surface-to-surface distance S, as a function of the annealing temperature T. Continuous lines are theoretical fits.

Five experimental values for R and S are reported as functions of T in Fig. 5. The continuous lines are the fits by Eqs 3 and 4. *E*_*A*_ = 0.227 eV, A = 376.156 nm and B = 262.225 nm are obtained *via* those fits. We noticed the diffusion activation energy of Ag/SiO_2_ annealed in our gaseous environment (*E*_*A*_ = 0.227 eV) was lower than that reported in a literature for surface diffusion activation energy of silver thin films in a vacuum ∼0.3–0.44 eV^34^. The deviation between our data and the literature is due to the difference in gaseous environment. Gaseous environment has a great influence on the morphology evolution of AgNIs^35^. The activation energy for the diffusion process is the potential energy barrier to diffusion, which indicates the minimum energy is required to start the diffusion. The activation energy for the diffusion process is related to the characteristics of substrate surface in the surface diffusion process. The characteristics of substrate surface are affected by its surrounding environment. Thus, the activation energy is affected by its surrounding environment. Flowing mixed gases of *H*_2_ (40 sccm) and *N*_2_ (200 sccm) are applied in our annealing process, which may result in the lower diffusion activation energy than vacuum environment.

The values of three constants are chosen to carry out the quantitative analysis. Based on these data, we can obtain the following quantized relationship:

5 $$R(T)=376.156\,{\exp }(-\frac{438.564}{{T}})$$

6 $${S}({T})=262.225{\exp }(-\frac{438.564}{{T}})$$

As for the contact angle, we use the energy relationship to study it. For the dewetting of Ag film, it is a thermally activated process reducing the free energy of the system by rearranging the film surface, the interface between the film and substrate, and the grain boundaries within the film. Without elastic strain, Young’s relationship should be satisfied for an island in an equilibrium on a planar substrate^28^.

7 $${{\gamma }}_{{mv}}-{{\gamma }}_{{s}\upsilon }={{\gamma }}_{{fs}}{\rm{c}}{\rm{o}}{\rm{s}}{\theta }$$

where *θ* is the contact angle, ${\gamma }_{{fs}}$ is the interface energy between the film and substrate, ${\gamma }_{{m}\upsilon }$ and ${{\gamma }}_{{sv}}$ are the surface energies of the metal film and the SiO_2_ substrate, respectively. The ${{\gamma }}_{{fs}}$, ${{\gamma }}_{{mv}}$ and ${{\gamma }}_{{sv}}$ are inversely proportional to the temperature, but $({{\gamma }}_{{mv}}-{{\gamma }}_{{sv}})/{{\gamma }}_{{fs}}$ (contact angle) is almost independent with temperature^33^. Thus, we can regard the contact angle as a constant in our analysis.

**Figure 6:**
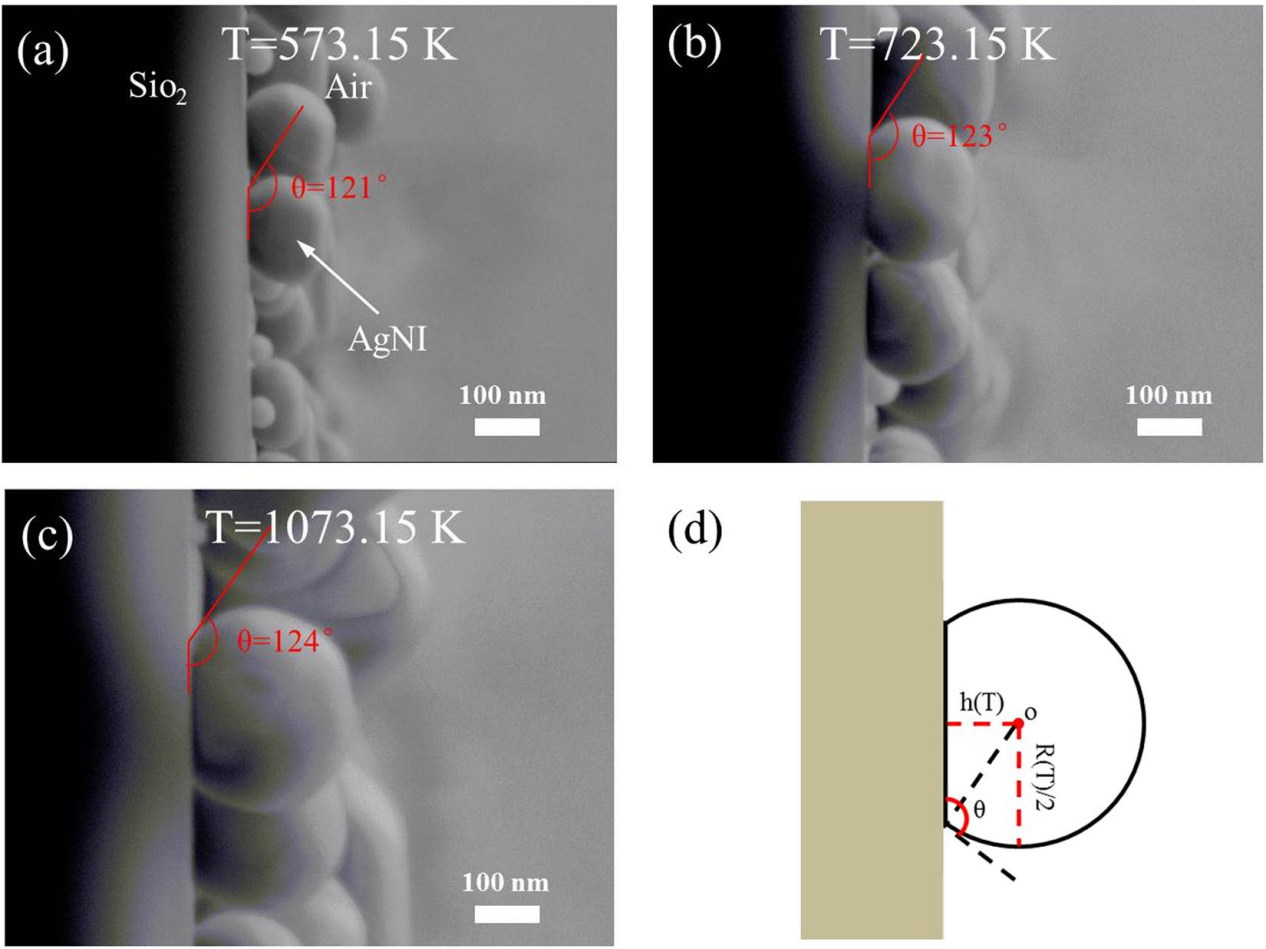
(**a–c**) The side-view images of AgNIs annealed at 573.15 K, 723.15 K and 1073.15 K, (**d**) the geometric reconstruction of AgNIs.

 Fig. 6a∼c show the side-view images of AgNIs annealed at 573.15 K, 723.15 K and 1073.15 K, and indicate the contact angles of AgNIs are 121°, 123° and 124°, respectively. We noticed there was little difference among those contact angles, which confirmed our conclusion that the contact angle can be regarded as a constant in our analysis. Meanwhile, we found all contact angles of our samples were around 123°. Thus, we set all contact angles in the following analysis to 123°. Based on the geometric reconstruction in Fig. 6d, we get the following equation:

8 $${h}({T})={\rm{s}}{\rm{i}}{\rm{n}}(33^\circ )\frac{{R}({T})}{2}$$

Based on the Eqs 5, 6 and 8, the temperature-dependent growth of AgNIs is quantitatively evaluated in SLDP. Concerning the temperature-dependent growth of AgNIs estimated in above work, the following observations are worth of mentioning:

- The temperature-dependent growth of AgNIs was quantized by Eqs 2 and 3 with fixed experimental conditions. Fixed experimental conditions include fixed Ag film thickness, fixed annealing ambiences, fixed annealing time, fixed substrate characteristics and so on. Different experimental conditions would change some parameters above. Although those changes can associate with one or more constants above *via* function, it would not be discussed in this work.
- Those equations are just suitable for the dewetting of Ag film in SLDP, which is from 573.15 k to 1073.15 k in our work. As for other annealing temperatures, our quantitative analysis is not applicable, due to the different principle in morphology evolution of AgNIs.

Besides, we conclude that, on a planar substrate, any temperature-dependent growth of metal nano-islands can be fitted by the equation in a certain temperature range with three to five samples which are prepared by the different dewetting temperatures. More importantly, if we know the surface diffusion activation energy on the metal film-substrate interface in advance, only one sample is needed to get quantitative information about the morphology evolution of metal nano-island as a function of the annealing temperature. For example, if we have gotten the activation energy coefficient of fixed interface, we could apply this activation energy coefficient to other substrates with only thickness of the metal film varies and use one sample to get quantitative information about the morphology evolution of metal nano-island as a function of the annealing temperature.

### Simulation of electromagnetic Field

To investigate the correlation between the LSPR effect and the dewetting temperature of Ag film, the spatial distributions of electromagnetic field intensity were simulated with the finite element method simulations (COMSOL Multiphysics). The incident light (633 nm) with X-polarization traveled along the - Z-direction. The optical properties of Ag were taken from Palik, and the refractive index was set to −14.461–1.1936·i corresponding to the incident wavelength^36^. AgNIs were set as spherical caps with a fixed contact angle (123°). The distribution of AgNIs was arrayed with 3 × 3. The spacing and diameter of AgNIs were set as the function of the dewetting temperature *via* Eqs 5 and 6, respectively. All the parameters are shown in Table 2.

**Table 2:** The geometric parameters used in the simulation.

T (K)	R (nm)	S (nm)	h (nm)
573.15	175.01	122.00	49.31
623.15	186.09	129.73	52.433
673.15	196.07	136.69	55.246
723.15	205.11	142.98	57.792
773.15	213.31	148.70	60.104
823.15	220.79	153.92	62.211
873.15	227.63	158.69	64.138
923.15	233.91	163.06	65.907
973.15	239.69	167.09	67.535
1023.15	245.03	170.81	69.038
1073.15	249.97	174.26	70.432

**Figure 7:**
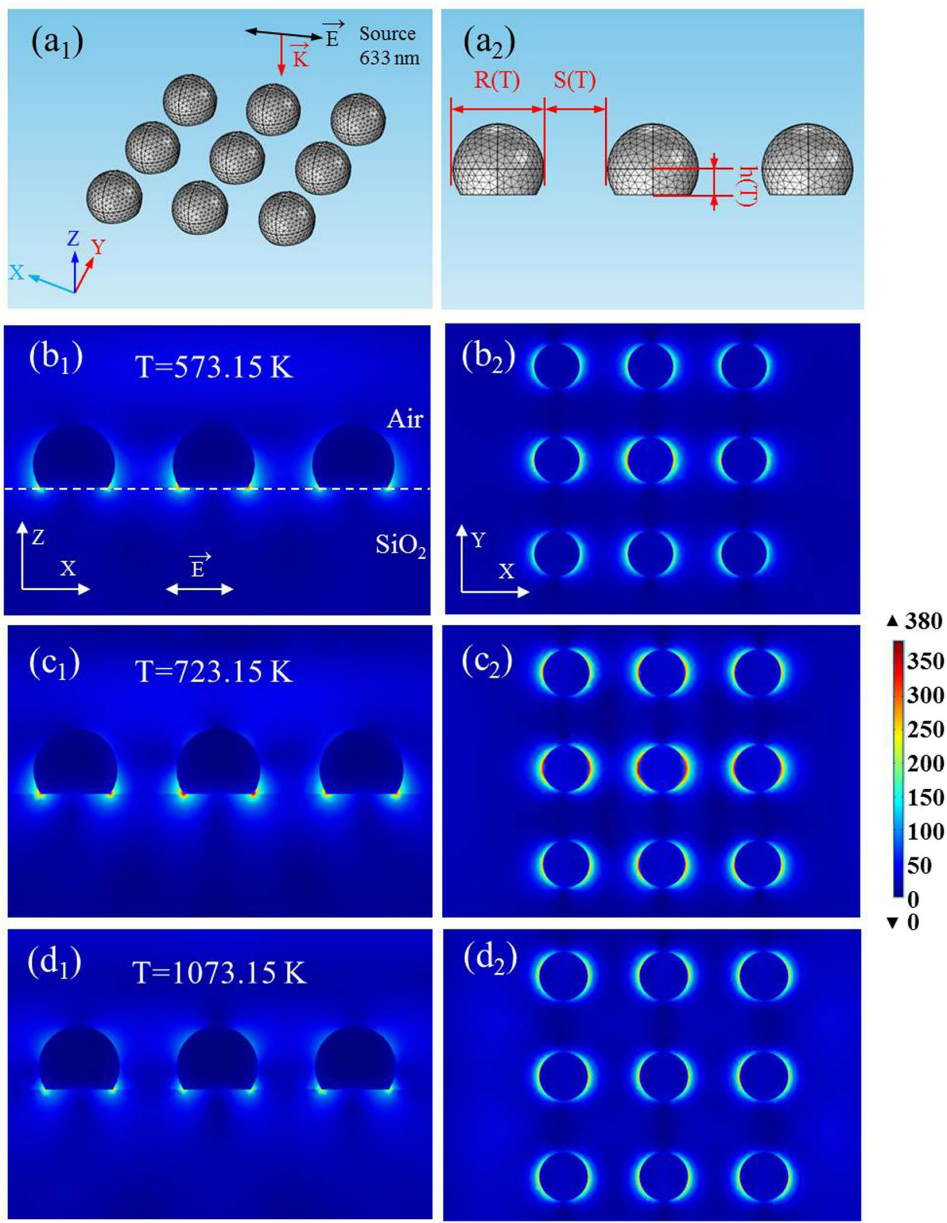
Simulation analysis of (**a**) schematic model, (*b*_1_, *c*_1_, *d*_1_) E-field distribution in x-z plane for the samples annealing at different temperature, (*b*_2_, *c*_2_, *d*_2_) E-field distribution in x-y plane for the samples corresponds to *b*_1_-*d*_1_, respectively.

 Fig. 7b∼d show the electric field distribution of AgNIs at 523.15 K, 723.15 K and 1023.15 K with different sections, respectively. We noticed all the locations of “hot spots” excited by AgNIs in different temperatures were located in the junction among air, substrate and AgNIs. The “Hot spots” excited by AgNIs in different temperatures only show obvious difference in intensity. Meanwhile, Fig. 7b indicates that a part of electric field is coupled into SiO_2_. In SERS test, probe molecules actually can’t enter into the SiO_2_, which means the electric field (energy) in SiO_2_ is wasted. Thus, for those substrates, only the electric field in air is effective in SERS.

**Figure 8:**
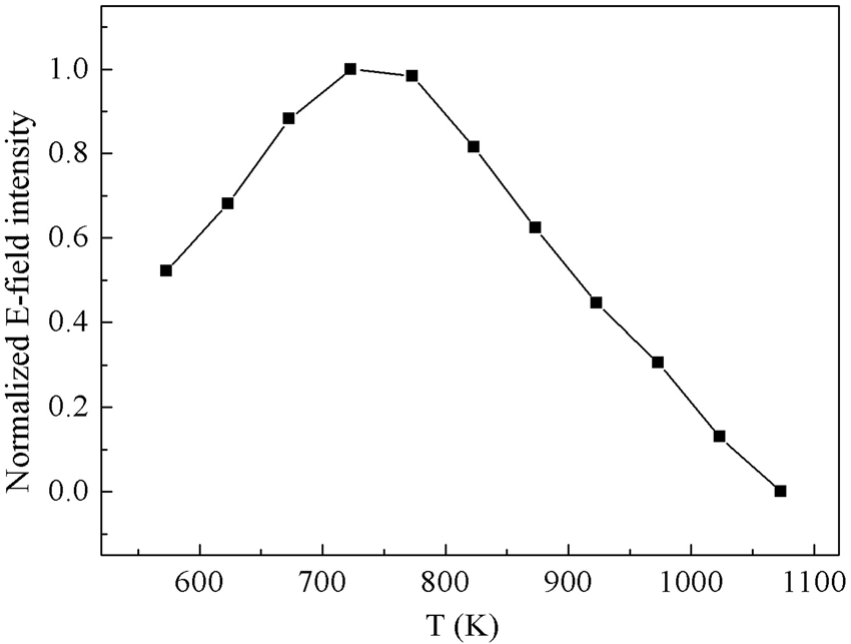
Normalized intensity of simulation as functions of T.

To evaluate the electric field enhancement of substrate, the averaged four power of the electric field around effective area of electric field enhanced on per unit area (${{E}}_{\upsilon }^{4}$) was simulated out. The normalized intensity of ${{E}}_{\upsilon }^{4}$ is shown in Fig. 8 as the function of the dewetting temperature. With the increase of temperature, the normalized intensity of ${{E}}_{\upsilon }^{4}$ increases firstly and then decreases. The maximum intensity of ${{E}}_{\upsilon }^{4}$ occurs in 723.15 K.

### SERS test

**Figure 9:**
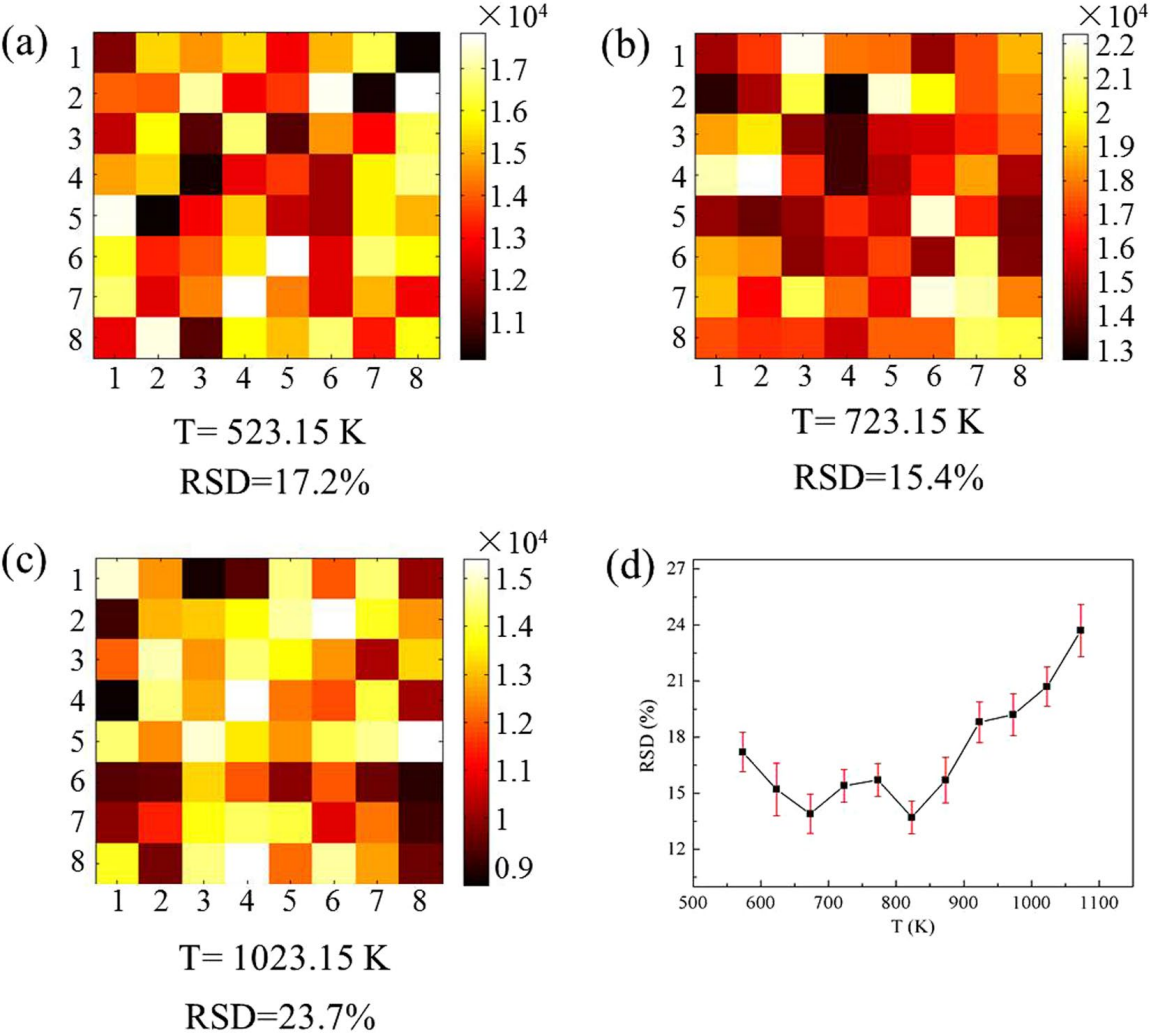
Raman intensity mapping of AgNIs annealed at 1509 *cm* ^−1^ for samples annealed at (**a**) 573.15 K, (**b**) 723.15 K, and 1073 K; (**d**) RSDs of Raman intensity mapping at different temperatures in our experiments, with the standard deviation shown by the error bars.

To investigate the SERS activities of our samples annealed at different temperature within 573.15∼1073.15 K, we took a Raman mapping on an area of 16 ×16 *μm* ^2^ for 10^−6^ M R6G. Fig. 9a∼c display the spot-to-spot SERS mappings at some representative temperatures, which was recorded with a step of 2 *μ*m using the integrated area of the baseline-corrected peaks at 1509 cm^−1^. The SERS enhancement factor (EF) of AgNIs at 1509 cm^−1^ was calculated based on the mapping data^37,38^. The results showed that the EF of AgNIs is in the range from 5 × 10^5^ to 1.1 × 10^6^. Besides, the RSDs of each mapping in SERS intensities at 1509 cm^−1^ were calculated and shown in Fig. 9d, with the standard deviation shown by the error bars. The RSD values and the standard deviation of RSD reveal that the SERS test of AgNIs is in trouble of reproducibility and repeatability, respectively.

**Figure 10:**
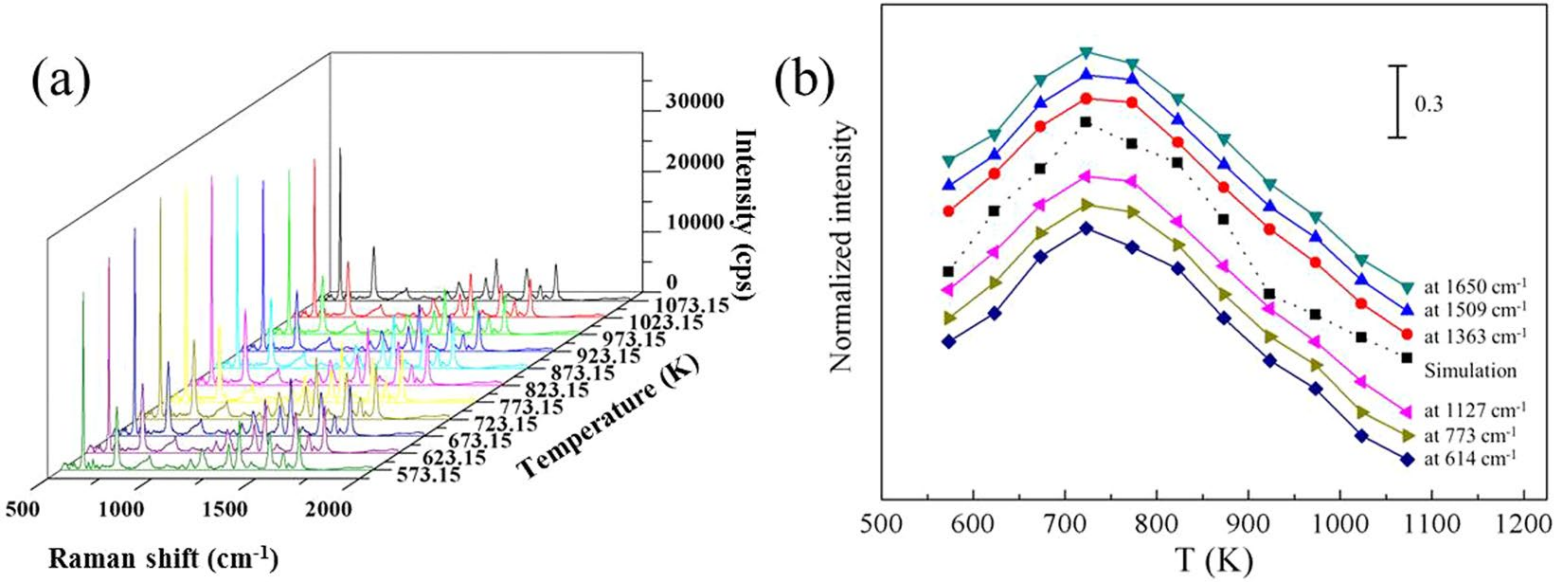
(**a**) Raman mapping data of AgNIs at different temperature, and (**b**) normalized intensity of SERS intensity at different characteristic peaks as functions of T, the simulation result is also given.

The trouble of reproducibility and repeatability make it difficult to complete a comparative analysis directly. Thus, the average processing is used to weaken the influence of reproducibility and repeatability. For further averaged data process, at each temperature, three samples from different batches were used for further testing. We average the 192 baseline-corrected spectral data sets from three samples prepared by the same annealing temperature and the resulting averaged spectra at different temperatures are shown in Fig. 10a. A plot of normalized SERS intensity at different characteristic peaks of R6G as a function of the annealing temperature is shown in Fig. 10b. Each data point represents the normalized average intensity.

From Fig. 10b, we noticed that the normalized average SERS intensity increased firstly and then decreased, and the maximum intensity occurred in 723.15 K. The experimental and simulation results show a good consistency on the trend with small deviation, which clearly indicates that our approach can qualitatively estimate the correlation between the dewetting temperature of metal film and SERS intensity in SLDP. As for these deviations between experimental and simulation results, it cannot be ignored and totally eliminated, but it is acceptable. These deviations can be attributed to the combination of many factors. To make sure the deviation within the acceptable range, which means that our experiments and simulations could have a good consistency, we have done a lot of work to optimize the experiments and analysis process. We further describe the main cause of deviation and corresponding solution as follows.

1. Inherent instability of SERS test. The trouble of reproducibility and repeatability is inevitable in the SERS test. To reduce the influences of reproducibility and repeatability on our analysis, all the data used in the analysis are the averaged values of mapping data from the different batches of samples, which are annealed at the same condition. Thus, the repeatability and reproducibility has a great influence on SERS quantification, but only brings a very small amount of deviation deviations to our analysis.
2. Fitting error. We get the values of R and S *via* the function fitting. Thus, the quantitative information about the morphology evolution of AgNIs in SLDP is not a perfect value, which inevitably introduces some fitting errors. Thus, in our analysis, to control the fitting error within an acceptable range, we generally use five samples to obtain quantitative information about the morphology evolution of AgNIs in SLDP. Although less than five samples could also get the quantitative information about the morphology evolution of AgNIs in SLDP, there may be more deviations when we obtain the correlation between the dewetting temperature of metal film and SERS intensity, especially at maximum position.
3. Fluctuations in experimental conditions. Our experiments cannot guarantee that the experimental process is completely identical, which has some fluctuations in the gas environment, temperature change rate, metal film quality and so on. Fortunately, the influence of fluctuations in experimental conditions has been greatly reduced on our analysis by the average effect. Meanwhile, we try our best to ensure the consistency of experimental conditions including the heating and cooling rate, gas environment, sample placement in furnace and so on.
4. The difference in the number of analyte molecules in the enhancement region on different substrates. The SERS intensity is essentially dependent on both the electromagnetic field and the number of adsorbed molecules in the enhancement region. The simulation of electromagnetic field just indicates the changing in the electromagnetic field, which is a dominant reason to change in SERS signal^39^. The number of adsorbed molecules in the enhancement region will also give a change in SERS signal. For this problem, we prepare the samples for SERS measurements *via* saturated adsorption of probe molecules on the substrate. Firstly, we soak the substrate in the solution identical 10^−6^ M R6G aqueous solution (30 mL) for 3 h. Subsequently, all the samples were rinsed with deionized water and dried by nitrogen gas flow. Therefore, we could make sure the surface especially the “hot spots” adsorbed a layer and only a layer of R6G molecules.

**Figure 11:**
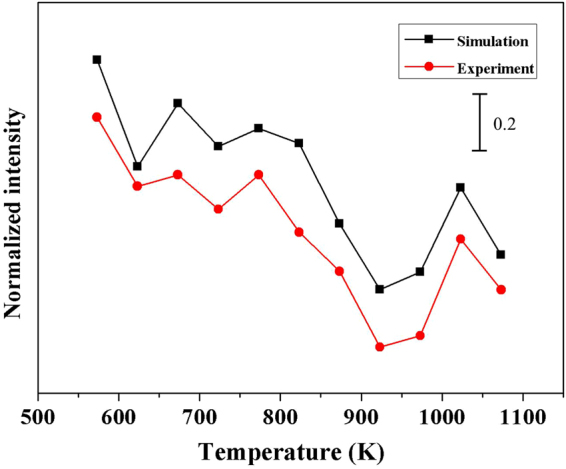
Normalized intensity of SERS intensity and simulation results as functions of T for 15 nm Ag film.

Thus, we conclude that, once the temperature-dependent growth of metal nano-islands is fitted by Eqs 5, 6 and 8 *via* five samples, we can obtain the changing trend of SERS intensity as a function of the annealing temperature with an acceptable deviation in the temperature range of SRTP. When applying the thermally dewetted metal nano-islands in SERS, we can determine an optimal dewetting temperature based on the comprehensive demand of nanostructures and SERS signal. Moreover, to demonstrate that our method is feasible to different metal film thicknesses, we used our analytical method in 15 nm Ag film to investigate the correlation between the dewetting temperature of metal film and SERS intensity, and the results were shown in Fig. 11. We noticed that the experimental and simulation results also showed a good consistency on the trend.

## Discussion

In conclusion, we have demonstrated a strategy for investigating the correlation between the dewetting temperature of Ag film and SERS intensity. We used a small amount of samples prepared by different dewetting temperatures to get the quantitative information about the morphology evolution of AgNIs as a function of the annealing temperature in SLDP. Those functions were then used to successfully obtain the correlation between the dewetting temperature of Ag film and SERS intensity *via* the simulation of electromagnetic field. When applying the dewetted AgNIs in SERS, our work can be used to determine an optimized dewetting temperature based on the comprehensive demand of nanostructures and SERS. This work could be applicable for different materials of metal film or planar substrate. Moreover, if we use more complex equations to import the effects of other parameters, including film thickness, pressure, and underlying substrates, based on our current work, it will be more flexible for the application of the dewetted nano-islands in SERS.

## Methods

### Preparation of AgNIs

Ag films with the thickness of 20 nm and 15 nm were deposited onto SiO_2_ substrates at a rate of about 1Å s^−1^ and at a pressure of about 10^−3^ pa by thermal evaporation. Dewetting of Ag film was performed in a quartz tube furnace with flowing mixed gas of *H*_2_ (40 sccm) and *N*_2_ (200 sccm). We divide the whole annealing process into four stages. Firstly, the furnace temperature rises from room temperature to 473.15 K with the heating rate of 10 K/min. This stage aims to reduce the influence caused by the instability of quartz tube furnace in low temperature. Secondly, the furnace temperature rises from 473.15 K to the dewetting temperature with heating rate 15 K/min. Thirdly, the furnace is kept at the dewetting temperature for 1 h to complete the dewetting process. Fourthly, the furnace temperature is cooled down naturally. *H*_2_ was introduced into the furnace to remove the metal oxides.

### Preparation of samples for SERS measurement

Rhodamine 6 G (R6G) was used as SERS probe molecule. The samples for SERS measurements were prepared by immersion of the substrates in an identical 10^−6^ M R6G aqueous solution (30 mL) for 3 h to make sure the surface especially the “hot spots” adsorbed a layer of R6G molecules. Subsequently, all the samples were rinsed with deionized water and dried by nitrogen gas flow.

### Characterization and SERS measurement

The morphology of samples was characterized by field emission scanning electron microscopy (SEM, JEOL, JSL-7800F). Raman spectra were recorded with a laser confocal Raman spectrometer (Horbia Jobin Yvon LabRAM HR Evolution) equipped with a 50× objective lens (numerical aperture (NA) of 0.75), and work distance (WD) of 0.37 mm. A 633 nm He-Ne laser with a power of 17 mW (filter of 5%) and a grating of 600 g mm^−1^ was chosen for measurements. The laser spot area was about 1 *μ*m *times* 1 *μ*m, and an integration time of 1 s was used in the measurements to reduce the heating effect induced by laser.
